# “A way to think of the client holistically”: Factors influencing speech-language pathology students’ ICF regard and uptake

**DOI:** 10.15694/mep.2019.000061.2

**Published:** 2019-12-16

**Authors:** Ingrid Scholten, Kate Ross, Jane Bickford

**Affiliations:** 1 Flinders University

**Keywords:** biopsychosocial, health professions, ICF, mixed methods, speech-language pathology, students, survey

## Abstract

This article was migrated. The article was marked as recommended.

Adoption of the International Classification of Functioning, Disability and Health (ICF) may facilitate holistic delivery of health and social care and improve interprofessional practice, however there is limited uptake across the spectrum of health professions, including speech-language pathology (SLP). Improved commitment will partially depend on student education, yet related educational research is scant.

Method

In order to inform teaching, learning and future research practices, this exploratory mixed methods investigation surveyed 101 student speech-language pathologists to describe how the ICF is regarded and used, and factors contributing to its acceptance.

Results

As with their professional colleagues, SLP student uptake of the ICF was limited. Those who used the ICF applied the framework and terminology alone, rather than its classification, coding or core set features, for client-centred rather than management tasks. Similarly, students appreciated the ICF for its ability to foster holistic practice, rather than its capacity to enhance workplace communication, a key factor in interprofessional practice. Statistical analysis of responses to scaled survey questions revealed the most valued learning experiences, especially case studies, lectures, ICF application in university assignments and on placement. Survey responses were significantly influenced by two factors: number of student placements and whether or not students had *only* a paediatric placement. Thematic analysis of open responses revealed two principal and one secondary theme: “ICF framework as a *way of thinking*”; “experiential learning optimises application of the ICF”; and “rudimentary understanding restricts ICF uptake”.

Discussion

Findings are discussed in relation to a proposed Transition from Theory to Practice model. Explicit integration of a biopsychosocial approach to practice across the curriculum should result in deeper understanding of the ICF, increased ability to apply it to interprofessional practice and, importantly, a greater sense of agency to effect change.

## Introduction

The International Classification of Functioning, Disability and Health (ICF) is a universal framework and classification system with multiple applications, including as a clinical tool, for managing statistics, research, social policy and in education ( World Health Organisation, 2013). The ICF supports person-centred practice and promotes a biopsychosocial model of care ( World Health Organisation, 2013), encouraging consideration of medical, social, and individual aspects of a person in relation to their health. Importantly, use of the ICF has the potential to enhance interprofessional practice through the use of a common language ( Allan *et al.*, 2006; Stephenson and Richardson, 2008). The key components of the framework are simple: *Body structure* refers to anatomical body parts and *body functions* refersto physiological and psychological functions. *Activities* are tasks executed by the individual and *participation* refers to that individual’s involvement in life situations. Contextual factors include *environmental factors*,such as social structures and family situations, that may facilitate or cause barriers for that person’s functioning, and *personal factors,* including personality, coping style and overall behaviour pattern ( WHO, 2013). Each ICF component has an opposite counterpart: impairment, activity limitations and participation restrictions respectively. The ICF differs from its predecessor, the International Classification of Impairment, Disabilities and Handicaps (ICIDH), as it integrates impairment with a person’s functioning, participation, and contextual factors rather than following the earlier disconnected and impairment focused view ( WHO, 1980).

While there is widespread acceptance of the ICF ( Jelsma, 2009), limited and inconsistent uptake across the spectrum of health professions is reported (e.g. Darrah, 2008; Peters-Brinkerhoff, 2016; Ross, Bickford and Scholten, 2018; Stewart *et al.*, 2013). A cultural change from the traditional biomedical model towards more holistic consideration of functioning and disability is likely to depend in part on student education ( Bornbaum *et al.*, 2015). Embedding learning more broadly across a program’s curriculum might be most conducive to supporting a paradigm shift from a biomedical to the biopsychosocial model of healthcare ( Stallinga *et al.*, 2018). There are examples and theoretical discussions regarding such integration within health professions curricula ( Cockburn, Trentham and Kirsh, 2005; Darrah *et al.*, 2006; de Brouwer *et al.*, 2017; Jones, 2011; Ottenbacher, 2000; Reed *et al*., 2008; Skarakis-Doyle and Doyle, 2008 and Sandborgh *et al.*, 2018). Health professions’ accreditation bodies typically stipulate inclusion of the ICF in university curricula to meet professional standards. However, ICF implementation or evaluation in higher education has received limited examination ( Bornbaum *et al.*, 2015) and the extent to which this is enforced is not known.

In conclusion, health professional accreditation standards typically specify embedding the ICF framework in practice, in keeping with its intended universal application. However, health professionals across jurisdictions demonstrate inconsistent and restricted uptake, and ICF education appears to be variable. A cultural change from the traditional biomedical model to a more holistic way of practising is likely to depend in part on student education, yet research in this arena is sparse, with insubstantial evidence about effectiveness.

In order to inform educational practices related to the ICF this exploratory study aimed to describe how the ICF is regarded and used by one group of students, speech-language pathologists (SLPs), including what learning approaches, experiences and resources are valued and factors contributing to uptake.

## Methods

Return of the online questionnaire, supported by LimeSurvey ( Schmitz, 2015) implied consent to participate. All currently enrolled senior Australian student SLPs in entry-level programs (3 ^rd^ and 4 ^th^ year undergraduates and all masters’ students) were eligible to contribute, with recruitment occurring primarily via invitation from a professional association newsletter and program websites.

**Research Instrument**. An existing survey ( Stewart *et al.*, 2013) was adapted, with permission, to reduce redundancy, improve data retrieval processes, and add literature-supported detail regarding potential uses and benefits of the ICF. The revised questionnaire contained three main sections: I. Use/Application of the ICF, targeted familiarity, level of comfort, knowledge development and nature of use; II. Utility of the ICF, reflected perceived value, barriers and enablers related to use; and III. Demographics, provided students’ study-related details. Table 1 outlines questions for the first two sections and the full survey is available on request. Survey completion required 15-20 minutes.

**Table 1.** Survey questions

Section	Questions	Sub questions	Question type
ICF Use	Knowledge level	-	Likert scale
	Level of comfort	-	
	Knowledge Development	10	
	Use of Components	7	
	Adoption of ICF	24	
ICF Utility	Advantages and disadvantages	-	Open response
	Perceptions on utility	10	Likert scale
	Barriers and enablers	-	Open response

**Statistical Analysis**. Statistical analysis of multiple choice, categorical and scale questions involved descriptive statistics, Chi-square goodness of fit to check for sample bias, normality testing of interval data to inform inferential testing, and exploratory factor analysis. Partial data were used where possible; data were analysed using IBM SPSS (IBM Corporation, 2015). Significance was established at a p-value of less than .05. Effect sizes were calculated for all inferential tests.

**Thematic Analysis.** Thematic analysis ( Braun and Clarke, 2006) of open responses was conducted via the lens of familiarity, with measures taken to enhance rigour. The researchers’ familiarity with the ICF and related literature guided initial coding, although we remained receptive to new ideas that emerged from the data. Two researchers were experienced with qualitative methodology. Analysis was aided by NVivo software ( QSR International, 2015). Memos were written during the analytic process to further develop interpretations and understanding. All researchers read responses multiple times to enhance familiarity with the content. One researcher (IS) coded the data using a recursive style. Initially line-by-line coding proceeded as the researcher asked “ *What is happening here*?” A process of constant comparison of codes occurred such that open codes were linked to develop emerging categories which were shared and discussed with the team. In the final stage of selective coding these categories were grouped to form themes. A second researcher (JB) coded 20% of the data independently and following further team discussion all responses were re-coded using the derived coding scheme.

## Results/Analysis

Responses were received from 101 student SLPs, with 79 full responses (all female) and 22 partial responses (typically missing demographic information).

**Demographics**. Demographic data included gender, study program (bachelor or master’s), and clinical placement factors. See Table 2.

**Table 2.** Demographics

		N	Sample %	CI
Gender	Female	79	78.2.%	69.2 - 85.2
	Male	0	0.0%	0.0 - 0.37
	Not Specified	22	21.8%	-
	Total	10179 ^a^	100.%	
Program of Study	Bachelors	40	39.6%	30.6 - 49.3
	Masters	39	38.6%	29.7 - 48.3
	Not Specified	22	21.8%	-
	Total	101	100%	
Number of Placements	0	4	3.9%	1.5 - 9.7
	1	10	9.9%	5.5 - 17.3
	2	20	19.8%	13.2 - 28.6
	3	30	29.7%	21.7 - 39.2
	4	14	13.9%	8.4 - 21.9
	Not Specified	23	22.8%	-
	Total	101	100.%	
Area of Practice	Paediatric focus	20	19.8%	13.2 - 28.6
	Adult focus	18	17.8%	11.6 - 26.4
	Both	31	30.7%	22.5 - 40.3
	Not Specified	32 ^b^	31.7%	-
	Total	101	100.%	
Service Delivery Setting	Various child settings	15	14.9%	9.2 - 23.1
	Community centre/outpatient	11	10.9%	6.2 - 18.5
	Specialist clinics/university clinics	9	8.9%	4.8 - 16.1
	Acute and rehabilitation	15	14.9%	9.2 - 23.1
	Mixed settings	29	28.7%	20.8 - 38.2
	Not Specified	22	21.8%	-
	Total	101	100.%	

^a^ Confidence interval for population was calculated using 95% interval. Missing data were excluded from calculations.

^b^ A few respondents specified their area of experience (e.g. speech/language or multimodal communication) but did not indicate whether this was adult, paediatric or both. This was classified as missing data.

**Level and comfort of ICF knowledge.** Typically, student SLPs were generally confident about their ICF knowledge base and somewhat comfortable in its use, indicating “good” or better knowledge (max.=5); (n=101; x=3.30; SD=0.855) and comfort levels (n=101; x=3.47; SD=0.870).

**Knowledge development**. The most positively received aspects contributing to SLP students’ ICF learning included application of ICF to case studies (n =99; x = 3.8 [max.=4]; SD=1.016; [very helpful]); lectures (n=100; x = 3.8; SD=0.931 [very helpful]) and use of the ICF in assignments (n=99; x = 3.8; SD=1.072 [very helpful]). Student SLPs also found client contact and discussion with practice educators (PEs) to be very helpful (n=99; x = 3.6; SD=1.135) while discussion with peers was moderately helpful (n=99; x = 3.3; SD=1.034).

**Use of and regard for the ICF**. Following exploratory factor analysis, two factors were extracted for each of the scale question sections using the Kaiser Meyer Olkin (KMO) statistic, accounting for 56%, 62% and 49%of the variance respectively: *components* (Survey section I. 4); *adoption* (Survey section I. 5); and *utility* (Survey section II. 2).

***Use of ICF*** ***components.*** The two factors obtained for ICF components used were *General Framework* and *Coding.* Overall reported use was low; the most frequently used ICF components fall within the General Framework factor (the framework itself [n=98; x=1.82; max.=4; SD=1.287] and terminology [n=100; x=1.58; max.=4; SD=1.257]); fewer respondents used coding or core set aspects of the ICF (n=98; x=0.57 [max.=4]; SD=1.065).

***Adoption*** **.** The extent to which SLP students use the ICF is consistent with the two derived factors of *Clinical Focus* and *Service/Management Focus*, with more positive responses (max. 4) provided in the context of tasks with a clinical focus, such as: *goal-setting* (n=94; x=2.96; SD=1.100); *describing activities and participation* (n= 96; x=2.65; SD=1.098); *personal factors* (n=95; x=2.65; SD=1.120); *environmental factors* (n=96; x=2.5; SD=1.081); or as a *guide to selection of assessment tools* (n=95; x=2.31; SD=1.202). Conversely, the ICF was applied less frequently in management tasks: *supporting service integration and management* (n=94; x=2.12; SD=1.283); *assessing service quality* (n=94; x=2.04; SD=1.341); *assisting planning at a service level* (n=93; x=1.85; SD=1.132); or *establishing eligibility for services* (n=93; x=1.54; SD=1.274). Over 40% (n=39; 42.4%) of respondents indicated that it was either not applicable or they never used the ICF for *evaluation of therapy outcomes,* which also loaded onto the management factor.

***Perceived Utility of the ICF*** *.* Respondents indicated their regard for the ICF across multiple tasks. Factor Analysis supported two underlying dimensions, *Person-centred thinking* and *Enhances communication.*Student SLPs deemed the ICF promotes person-centred thinking (max. 5): *encourages holistic thinking* (n=81; x=4.62; SD=0.621); *fosters clinical reasoning and decision-making* (n=81; x=4.19; SD=0.754); *has great benefits* (n=80; x=4.3; SD=0.973); and *enhances ethical service provision* (n=81; x=3.92; SD=0.755). However, respondents generally had more neutral views regarding many of the variables associated with the *Enhances communication* factor, namely whether the ICF helps *clarify team roles* (n=80; x=3.13; SD=0.848); or *fosters teamwork* (n=80; x=3.18; SD=0.786).

**Trends in uptake.** There were no significant differences for any factors in relation to study program or previous study, nor regarding respondents’ knowledge confidence or level of comfort with respect to their placement settings or regarding areas of practice. Likewise, respondents’ knowledge confidence and ICF component use were unaffected by their number of placements. However, respondents’ placement experiences did influence a range of factors, with small to medium effect sizes: Student SLPs’ level of comfort with the ICF (p = .046, d = .232), application of the ICF to person-centred thinking (p = .005, d = .323), clinical focus (p = .005; d = .327) and service and management focus (p = .009; d = .306) all increased with two or more placements.

There were also significant differences for clinical focus in relation to area of practice (paediatric, adult or both; p = .006); post hoc testing revealed that respondents with *only* a paediatric placement indicated lower levels of ICF application for tasks with a clinical focus compared with respondents who had both adult and paediatric experience (p = .02).

**Figure 1.**
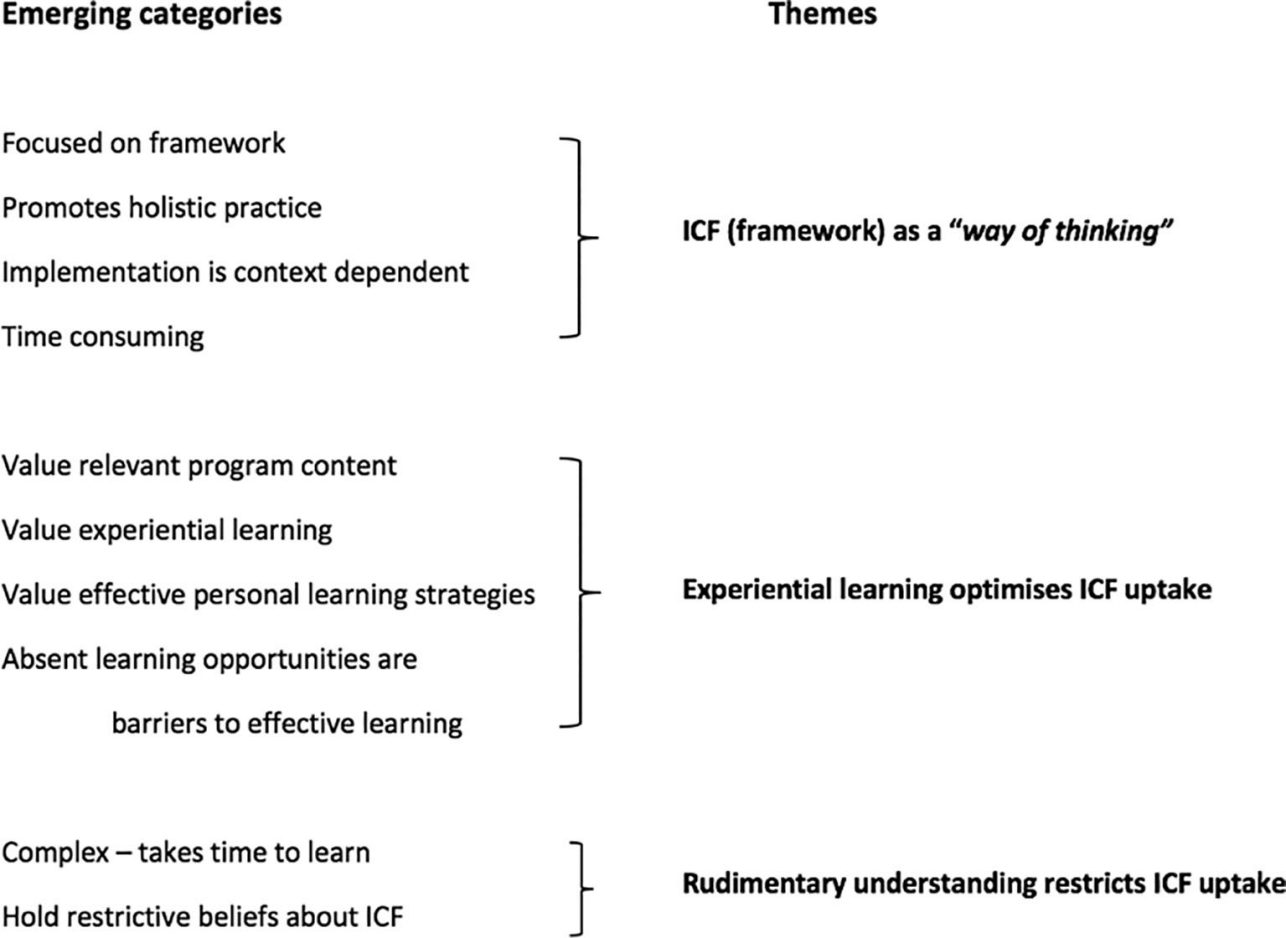
Coding tree: emerging categories and themes

**Thematic Analysis.** Eighty five SLP students responded to three open survey questions (most influential ICF factors; advantages and disadvantages; barriers and facilitators). Comments ranged from 1 - 143 words (mean = 27.1). Thematic analysis revealed two principal and one secondary theme: ICF (framework) as a “ *way of thinking*”; experiential learning optimises ICF uptake; and rudimentary understanding restricts ICF uptake (See Fig. 1). See Appendix for sample quotes according to theme.

**ICF (framework) as a “ *way of thinking”.*** It is clear that with few exceptions SLP students equate “the ICF” with the conceptual framework, particularly the activity and participation components, rather than its classification or coding features. The ICF model was used as a loose guide rather than overtly applied. Respondents recognised the ICF as guiding holistic practice. The biopsychosocial approach to care was perceived to lead to improved client outcomes, by guiding goal-setting and intervention. However, respondents also indicated that the ICF is infrequently used in specific settings or situations, in particular in acute hospitals and in paediatric practice. There may also be a lack of focus on the ICF in relation to the students’ other learning needs.

**Experiential learning optimises ICF uptake.** When commenting on factors that best enabled them to implement the ICF, respondents expressed their appreciation for relevant, clear and specific learning resources. They valued active learning experiences, both in the university setting and on placement. Both authentic and hypothetical case studies were highlighted as being particularly useful. Respondents mentioned the benefits of applying the ICF to university assessment tasks, although not all shared this majority view. Opportunities for discussion, with peers, lecturers, and PEs, and implementing the ICF on placement were valued.

While respondents appreciated the educational benefits of appropriate university experiences and placements, the absence of these was also noted. The reported top barriers to ICF implementation related to learning experiences, including a recognition of the respondent’s inadequate understanding of the ICF, theoretical lectures without clinical application, lack of workshops and tutorials, and limited opportunities while on placement. Few respondents commented on their own role in the learning process, mentioning the importance of actively seeking and working through relevant information or using specific strategies such as keeping a diagram of the ICF framework on hand.

**Rudimentary understanding restricts ICF uptake.** Some respondents are unaware of misunderstandings and restrictive attitudes that potentially limit further development of their ICF knowledge-base. For example, some respondents believe that the ICF must be applied in its entirety, that its use must always be explained or that only certain language can be used to describe it to clients.

The most commented on barrier to learning was the ICF’s complexity. Respondents described their frustration with the terminology, scope of the ICF and time required to “conquer” it. The distinction between activity and participation was especially problematic.

**Figure 2.**
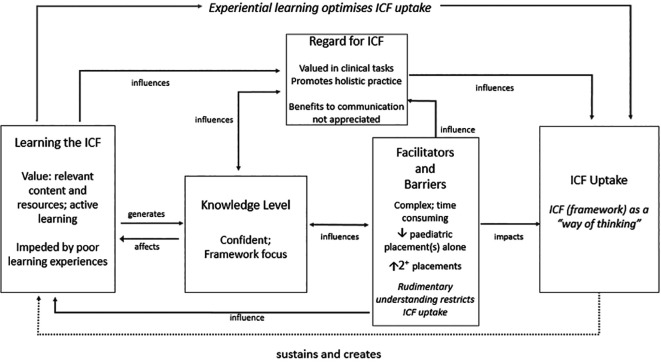
Transition from theory to practice (adapted from Ross, Scholten and Bickford, 2018)

**Model of ICF uptake.** Our combined statistical and thematic findings are in keeping with those relating to practitioners we described earlier ( Ross, Scholten and Bickford, 2018). Here we expound the model with specific details relating to student findings depicting the process of integrating the ICF into practice ( Figure 2). The cycle begins with learning experiences related to the ICF which generate knowledge and affect ICF regard and are in turn influenced by knowledge level, barriers and facilitators. Level of uptake, depicted on the far right of the model as using the ICF as a “ *way of thinking*”, is optimised by experiential learning. It is influenced by both regard for the ICF and facilitators and barriers, including the restrictive influences of rudimentary understanding. Practice and continued education sustain ICF use, closing the learning loop. Results that informed these interpretations are highlighted within the model.

## Discussion

Key findings of our investigation of how the ICF is regarded and used by student SLPs are organised and discussed here in relation to our proposed Transition from Theory to Practice model which implies where changes might be made to improve ICF uptake and adoption of a biopsychological approach to care.

**ICF (framework) as a “ *way of thinking*”.** Responses about SLP students’ ICF use and appreciation resembled those of health professionals reported earlier; typically the conceptual framework alone is applied to “document and guide clinical decision making” ( Darrah, 2008, p. 150). Similarly, SLP students use the ICF for clinical rather than management tasks.

Findings suggest that SLP students’ education has focused on the ICF framework, emphasising activity and participation, with less attention paid to other aspects. A review of Nordic papers also found “activity” was the most mentioned ICF component ( Maribo *et al.*, 2016). Creating effective change will demand specific attention to other ICF components, its benefits to professional communication, and its relevance at the societal level.

Student SLPs indicated that the ICF promotes holistic practice. While students and health practitioners value person-centeredness ( Bellon-Harn, Hartwell Azios and Dockens, 2017; Dockens, Bellon-Harn and Manchaiah, 2016), explicit person-centred interventions are uncommon ( Torrence *et al.*, 2016). Interventions typically address impairment and skill-based activity levels, focusing on tasks and target behaviours, rather than considering other ICF components, notably life participation ( Kjellberg *et al.*, 2012; Ramklass, 2015; Torrence *et al.*, 2016). Likewise, supervisory practices may target concrete, discipline-specific knowledge and skills at the expense of psychodynamic or intrapersonal factors ( DiLollo and Favreau, 2010).

In our study, SLP students did not identify the ICF as supporting workplace communication, either with same-profession colleagues or interprofessionally. The likely reason is that they are not typically working interprofessionally on placement ( Ramklass, 2015). Positive student experiences have been achieved by basing shared learning experiences around the ICF: by experienced PEs modelling interprofessional practice ( Snyman, Von Pressentin and Clarke, 2015), by following a simple structured tutorial process ( Cahill *et al.*, 2013), and holding regular interprofessional team discussions, carrying out joint treatment sessions and home visits, and socialising together ( Kloppers *et al.*, 2015). Learning occurs through interaction with others in a work context. It is important for students to “engage actively with the roles, beliefs, values and cultures of other professionals. Collaborative learning through tasks and discussions helps to achieve shared understanding and, in terms of clinical practice, shared goals” ( Thistlethwaite, 2012, p.65).

**Facilitators and barriers.** In this section we consider influences on ICF uptake: issues pertaining to practice settings and exposure; limited opportunity to bridge theory and practice; the time-consuming nature of ICF application; rudimentary understanding of the ICF; and the absence in the data of an appreciation of a broader perspective.

Whether or not SLP students had *only* a paediatric placement and the number of completed placements significantly influenced responses.

***Issues in Paediatric Practice***. Students’ views that ICF use is context dependent are consistent with those expressed by Australian SLPs ( Ross, Bickford and Scholten, 2018). If PEs aren’t using and discussing the ICF, students will not appreciate its relevance to practice. This reduced ICF adoption in paediatric settings may be partly because the paediatric perspective was more recently assimilated into the ICF ( World Health Organisation, 2012). In addition, developmental issues are underrepresented in the ICF coding structure ( Darrah, 2008). Castro and Palikara’s (2017) book about ICF implementation in educational and care systems may help to demonstrate the universal applicability of the ICF. However, a health-related framework may be considered irrelevant in disability and educational settings where different frameworks and priorities may apply.

***Improved ICF uptake with two or more placements***. The relationship between improved ICF uptake and two or more placements may pertain to SLP students’ continuing development of professional competency - a combination of increasingly demanding knowledge, skills and personal qualities ( McAllister *et al.*, 2011). A hierarchy of difﬁculty exists, with earlier development of easier competencies involving readily explained behaviours, such as punctuality or maintaining confidentiality, or those practised more frequently. More challenging competencies require complex cognitive skills such as data synthesis and analysis and interpretation of what this means for the client (ibid.). Initially, students are typically self-focused, gradually attending more outwardly, including to their clients.

Therefore, one explanation for the finding is that, in spite of protestations that it is complex and difficult to learn, it is actually internalised by students as involving relatively basic concepts, especially when considered alongside the multifaceted and complex areas of professional practice. Hence it develops early in the learning trajectory, but only once students have established a level of comfort with the clinical process.

***Limited opportunity to bridge theory and practice.*** Student SLPs experienced an unsettling disconnection between their academic learning and practice experiences, consistent with our previous report of limited ICF uptake by practitioners ( Ross, Bickford and Scholten, 2018). To facilitate translation and broader implementation of the ICF, university educators could offer PEs training and skill development opportunities in relation to the ICF. Such training will increase awareness of what students are learning and reinforce professional practice guidelines. It may also motivate PEs to identify opportunities for students to apply the ICF on placement. A participatory design approach ( Appleby and Tempest, 2006) could also be used to further understand the barriers and facilitators to ICF use and support its implementation.

***ICF is time consuming to implement.*** Student SLPs’ concerns about the time consuming nature of using the ICF may contribute to their lack of emphasis on coding and classification elements, and is consistent with findings elsewhere ( Bornbaum *et al.*, 2015; Jelsma, 2009). As practitioners become more familiar with the ICF and tools are developed to streamline documentation, the process should become less demanding, especially when applying the framework alone.

***Rudimentary understanding restricts ICF uptake.*** Like their professional colleagues, student SLPs expressed confusion between the concepts of activity and participation ( Van de Velde and De Vriendt, 2018) which, paradoxically, were the most cited influential components. However, the ICF is unlikely to be more complex than other professional content which is grasped by all successful students. The perceived complexity and how the framework is taught appear to have resulted in some students holding limiting beliefs. They described having “mental blocks” about using the framework as well as clearly holding some inaccurate views that obstruct further learning. A strong curricular theme of reflective practice and the development of metacognition should help address students’ independent learning skills.

***Absence of a broader perspective***. A notable absence in SLP students’ responses was mention of the potential for the ICF to be used at the societal level, including reference to barriers. While the academic focus goes beyond student SLPs’ understanding of relevant underlying conditions and mastery of specific treatment techniques, more comprehensive perspectives may require additional attention. To facilitate graduates’ involvement in shaping health reform and environmental change at the societal level, consideration of the macro perspective must be clarified and clearly integrated into curricula.

The ICF framework itself is deceptively simple; however, it has multiple levels and uses. Ways to improve student learning are addressed within the context of the final theme below.

**Experiential learning optimises ICF uptake.** Student SLPs value much about their educational experiences in relation to acquiring ICF knowledge and skills and identified the absence of these features as obstacles to learning. In order to change professional behaviour, problematic issues must be addressed within student education.

***Curricular change***. A number of papers describe the adoption of the ICF as a framework for health professions curricula to promote a paradigm shift towards a biopsychosocial approach to care ( Bornbaum *et al.*, 2015; Nguyen *et al.*, 2016; Ottenbacher, 2000; Skarakis-Doyle and Doyle, 2008; Stephenson and Richardson, 2008). Findings suggest that graduates’ practice embodies ICF principles in the context of strong professional competencies ( Sandborgh *et al*, 2018).

Assuming a biopsychosocial perspective of the individual’s health condition is a key factor in adopting person-centred practice which is often overshadowed by social, economic, and political inequalities. Duchan (2014), Fleming-Castaldy (2015) and Ghul and Marsh (2013) describe taking a macro perspective in their teaching, sharing examples designed to develop students’ emancipatory skills. Students consider sociocultural and historical views of health and social care through the use of rich narratives, presented by clients and caregivers, case studies, historical documents, film, and students’ own stories. The aim is to exploit students’ personal reactions and experiences in order that they learn important professional perspectives, including appreciation of each person’s unique experience, leading to greater understanding of person-centeredness and, in particular, the complexity of barriers and enablers of participation.

Explicit adoption of key themes for curriculum reform, including a biopsychosocial approach, the ICF and an emancipatory orientation, in the context of harnessing features identified by students in the present study as enabling their learning, would help students develop their professional identity as powerful change agents.

***Learning through the use of clear tools and models.*** There are tools and conceptual models that foster ICF application skills to improve practice, including decision-making frameworks to enhance reflection and reasoning ( Atkinson and Nixon-Cave, 2011; Dal Bello-Haas, Wojkowski and Kho, 2015; Darrah *et al.*, 2006; Jarl and Ramstrand, 2018; Jelsma and Scott, 2011). Embedding the ICF framework into routine tasks, such as writing reports, determining appropriate intervention or conducting case presentations, should enhance acceptance of the ICF’s relevance to practice ( Yardley, Teunissen and Dornan, 2012).

**Limitations.** Study findings are exploratory given the low number of respondents, the fact that students’ ICF knowledge was not explicitly assessed, their level of interprofessional experience was not identified and the survey tool, although used previously, was not tested for psychometric properties. Also, there were no opportunities for more in-depth interaction with respondents to explore their perspectives. Likewise, students from a single health profession are represented, the placement structure for which likely differs from other student groups. However, the fact that issues related to ICF education and application have been described across health professions may support the generalisability of our findings.

## Conclusion

Since the ICF’s launch, increasing attention has been paid to the importance of taking a biopsychosocial approach to practice, but there is limited research related to educating future health professionals in its application. Student SLPs surveyed in this study reported a range of positive findings in relation to what they know and value with regards to the ICF. Namely, they recognised that the ICF has the capacity to promote holistic, person-centred practice; they are guided by aspects of the framework, especially activity and participation, for the purpose of enhancing client outcomes; and two or more placement experiences augment their ability to apply the ICF for a range of purposes. Conversely, there were aspects of the ICF and its application that were not as well understood, reflecting the emphasis of their learning experiences. For example, its suitability in acute medical and paediatric settings was questioned; classification and coding aspects of the ICF were regarded as onerous and less useful; the benefits of a shared language for interprofessional communication were not widely appreciated and a paediatric practicum alone did not contribute to improved ICF clinical application. Regrettably, students’ focus remained at the clinical level, with no one appreciating the capacity of the ICF to enable a broader perspective to be taken, even if only to comment on barriers that restrict such a viewpoint.

Student SLPs found learning and applying the ICF to be demanding and experienced a disconnection between academic and practice learning. We suggest explicitly incorporating a biopsychosocial approach to practice within and across health professions’ curricula, including active learning experiences, germane and transparent resources, and authentic assessment tasks, in both university and placement settings. In this way the ICF could be addressed iteratively and more comprehensively, with students being more likely to engage with an emancipatory framework. Targeted training and skill development related to the ICF should support practice educators to provide cohesive learning and enhanced appreciation of the ICF’s many benefits. Such measures should result in students having a deeper understanding of the ICF, increased ability to apply it interprofessionally and, importantly, have a greater sense of their own agency to effect change.

## Take Home Messages

• Student SLPs appreciate the ICF framework for its ability to foster holistic practice, applying its components loosely in order to improve client outcomes, in some settings more than others.
• Student SLPs find the ICF challenging to learn and implement.
• Active learning experiences, with specific, relevant and clear resources, are appreciated in both university and placement settings.
• Student SLPs experienced a disconnection between academic and practice learning. Targeted training and skill development related to the ICF should support PEs to provide cohesive learning experiences.
• Health professions’ curricula including the ICF and its broad applicability should consider an integrated and iterative approach.

## Notes On Contributors

**Dr. Ingrid Scholten** is an adjunct senior lecturer and researcher at Flinders University, South Australia.ORCID identifier: https://orcid.org/0000-0002-1505-3047

**Kate Ross** completed her honours thesis on the ICF at Flinders University, and nowworks as a speech-language pathologist in a paediatric clinic.

**Dr. Jane Bickford** is a senior lecturer and researcher within the Speech Pathology discipline at Flinders University. ORCID identifier: https://orcid.org/0000-0001-6080-4279

## Appendices

Sample quotes by theme.

ICF (framework) as a “way of thinking”.

It is not something I have used explicitly in practice, but learning about it has given me a backdrop of holistic thinking to bring to my clinical placements (ID20_BSP_4_Both) ^1^

As my confidence and independence in planning and providing holistic services to clients has grown, so has the influence of the activity/participation, environmental, and personal components in determining suitable assessments, therapy goals, environmental modifications, outcome measurements and appropriate referrals (ID15_BSP_3_Both).

During my first placement, ICF use was minimal as clinical practice was still new and there were other priorities initially e.g. talk to parents, ask case history questions (ID127_MSP_3).

Experiential learning optimises ICF uptake.

Lectures that provide overviews re assessments/interventions that are appropriate and relevant to each level of the ICF (ID51_MSP_2_Adult)

Journal articles that apply the framework to actual cases have helped me to get an idea of how it can be used (ID129_MSP_3_Both)

I think it’d be useful to learn more about how to apply the ICF to assessment and intervention rather than just identify the different aspects of the ICF in a client’s life. (ID127_MSP_3_unknown)

I found it really beneficial to my learning of the ICF to have case study based activities that require its integration e.g. a TBI client case from assessment to therapy. It made me much more confident using the ICF with real-world clients (ID81_BSP_3_Both)

Assignments specifically asking for assessment and intervention planning according to ICF framework (ID106_MSP_3_Both)

The incorporation of ICF into exams has really helped my learning because it forced me to become very familiar with the framework and to practice applying it to a theoretical client (ID2_BSP_3_Both)

VIVA examinations (ICF use included in marking criteria helped me structure and think holistically re client) (ID51_MSP_2_Adult)

Having the experience of applying the ICF to clients I have worked with on placement has helped to solidify my knowledge (ID2_BSP_3_Both)

Don’t see its use modelled by clinicians in practice, only lecturers at university (ID40_MSP_4_Both)

(PEs) don’t talk about it much - we learn about it academically but I don’t feel it is well integrated into our ... placements (ID132_MSP_2_Both).

Rudimentary understanding restricts ICF uptake.

I at first found the ICF so overwhelming I would purposely neglect to consult it. (ID155_BSP_3_Both)

It is quite an abstract framework and takes some time to grasp (ID32_MSP_4_Both)

Endnote

^1^ID is comprised of electronically assigned survey number, student’s program level, number of placements and whether placements have been paediatric, adult or both.
