# Supplementary material for: “A way to think of the client holistically”: Factors influencing speech-language pathology students’ ICF regard and uptake
Source: MedEdPublish (2016). 2019 Dec 16;8:61. Originally published 2019 Mar 19. [Version 2] doi: 10.15694/mep.2019.000061.2 (PMC10712644; doi:10.15694/mep.2019.000061.2)
Supplement: Peer review history

# Reviewer report: Reviewer response for version 2

**DOI:** 10.21956/mep.20239.r31373

**Published:** 2019-12-17

**Authors:** Ken Masters

**Affiliations:** 1 Sultan Qaboos University

This review has been migrated. The reviewer awarded 4 stars out of 5 Although I still feel that the scope and limitations of the population surveyed, and, therefore, the study’s broader applicability, should have been addressed in more detail, the authors have addressed the major concerns regarding the data, and so have created a useful paper.

Reviewer Expertise:

NA

No decision status is available

# Reviewer report: Reviewer response for version 2

**DOI:** 10.21956/mep.20239.r31374

**Published:** 2019-12-16

**Authors:** Felix Silwimba

**Affiliations:** 1 University of Lusaka

This review has been migrated. The reviewer awarded 5 stars out of 5 this is a very important article for learners to appreciate disability issues. it should be extended to other professions in the health arena. I learnt some good lessons.

Reviewer Expertise:

NA

No decision status is available

# Reviewer report: Reviewer response for version 2

**DOI:** 10.21956/mep.20239.r31375

**Published:** 2019-12-16

**Authors:** Trevor Gibbs

**Affiliations:** 1 AMEE

This review has been migrated. The reviewer awarded 4 stars out of 5 I appreciate the way that the authors have addressed the reviewers concerns and I now feel that this paper is now a very good and informative paper. Not only does it appear useful to SLP students, but I think it does show the transferability of international Classifications and what applies to these SLP students could be transferred to other health professionals programmes.I would recommend this paper to all who are involved in Interprofessional Learning

Reviewer Expertise:

NA

No decision status is available

# Reviewer report: Reviewer response for version 1

**DOI:** 10.21956/mep.18990.r27224

**Published:** 2019-08-13

**Authors:** Trevor Gibbs

**Affiliations:** 1 AMEE

This review has been migrated. The reviewer awarded 3 stars out of 5 An interesting, although a very long paper to read, relating ti a rather specific part of a curriculum. It was extensively researched and very well referenced which I felt reflected that this was a major part of one of the author's Masters thesis. The length and depth of the paper I feel draws the attention of the reader away from the main impact of the research and could be extensively shortened and summarised to highlight the value of the topic.I do however feel that the paper, its content and its extensive reference list are of interest to curricula planners within the area, although i suspect this group of educationalists are very specific.

Reviewer Expertise:

NA

No decision status is available

# Reviewer report: Reviewer response for version 1

**DOI:** 10.21956/mep.18990.r27223

**Published:** 2019-07-06

**Authors:** Ken Masters

**Affiliations:** 1 Sultan Qaboos University

This review has been migrated. The reviewer awarded 3 stars out of 5 An interesting paper examining the attitudes and experiences of speech-language pathology students in Australia towards the International Classification of Functioning, Disability and Health (ICF) model.The paper begins with a detailed literature review of experiences and uptake of the ICF in general. The study is well conducted, and the Discussion sufficiently detailed.Some issues with the paper:• For the uninitiated, it might have been a good idea to have a single paragraph giving some details about the ICF (its rationale, history and chief components). From the introduction, one learns only really that it is a biopsychosocial model of healthcare, but not much more.• Given that the study is of speech-language pathology students in Australia, it would be better if the title reflected that, rather than students in general.• Elsewhere in the paper, including in the Take-home Messages, this broader view is also implied (or would be inferred by readers), so the authors should be careful to ensure that their Discussion and comments are always only about speech-language pathology students in Australia.• While it might not be possible to give absolutely accurate numbers, it would be useful to have at least an approximate estimate of the total number of speech-language pathology students in Australia, so that we can have some sense of the response rate.• I think there is a real problem with the exclusion of so much data from Table 1. From a reading of Table 1, more than 20% of the forms were excluded from the analysis merely because they did not indicate their gender. If people elect to withhold their gender, this does not appear to be grounds for ignoring their responses, particularly as the number is so high, and then results in only one gender’s being represented. The authors should rather have inserted a row for “unknown” and ensured that those forms were also included. (I may be misunderstanding the table; it is just that all the other categories also add up to equal to or less than 79, so it appears that those students who did not state their gender automatically had their responses to the other questions ignored.) In the later statistics (outside the table), however, a much high n is cited, so then it would appear that those other forms were included in the data. Some important clarification on these numbers is required.• Similarly, “We considered 85 students’ open responses to three survey questions”. This may just be a matter of phrasing. Did only 85 students give open responses, or were only 85 students selected?• The Model in Figure 2 is useful, but it is unclear how much of the model is from Ross et al 2018, and how much has been contributed by this study. Perhaps the authors could produce two figures so that this could be clarified. (And “Ross et al 2018” should be written as “Ross, Bickford and Scholten, 2018” as it is elsewhere in the paper).So, an interesting study, but it does need some tidying up.

Reviewer Expertise:

NA

No decision status is available

# Reviewer report: Reviewer response for version 1

**DOI:** 10.21956/mep.18990.r27225

**Published:** 2019-03-21

**Authors:** Felix Silwimba

**Affiliations:** 1 University of Lusaka

This review has been migrated. The reviewer awarded 5 stars out of 5 This research paper clearly brings attention to very important aspect of understanding disability assessment and evaluation. it is a must read for all those educating healthcare professionals. by making use of its recommendations awareness on disability and ICF will be enhanced among healthcare professional learners to encourage interdisciplinary collaboration in the care of clients across disciplines. the researchers clearly identified their limitations in carrying out the study even then its findings are applicable anywhere.

Reviewer Expertise:

NA

No decision status is available
